# Ideas and Enhancements Related to Mobile Applications to Support Type 1 Diabetes

**DOI:** 10.2196/mhealth.2567

**Published:** 2013-07-25

**Authors:** Andy Pulman, Jacqui Taylor, Kathleen Galvin, Mike Masding

**Affiliations:** ^1^The School of Health & Social CareBournemouth UniversityBournemouthUnited Kingdom; ^2^Bournemouth UniversityBournemouthUnited Kingdom; ^3^Faculty of Health and Social CareUniversity of HullHullUnited Kingdom; ^4^Diabetes CentrePoole Hospital NHS Foundation TrustPooleUnited Kingdom

**Keywords:** patient education, type 1 diabetes, mobile, apps, sociotechnical design, lifeworld, humanising healthcare, patient voice, empathy, ideas, enhancements

## Abstract

**Background:**

Mobile devices have become increasingly important to young people who now use them to access a wide variety of health-related information. Research and policy related to the integration of health information and support with this technology do not effectively consider the viewpoint of a younger patient. Views of young people with type 1 diabetes are vital in developing quality services and improving their own health-related quality of life (HRQOL), yet research on their lifestyle and use of Web and mobile technology to support their condition and in non–health-related areas is sparse.

**Objective:**

To develop insight into young people with type 1 diabetes and their current use of Web and mobile technology and its potential impact on HRQOL. This can be achieved by constructing an in-depth picture of their day-to-day experiences from qualitative interviewing and exploring how they make use of technology in their lives and in relation to their condition and treatment. The goal was then to build something to help them, using the researcher’s technical expertise and seeking users’ opinions during the design and build, utilizing sociotechnical design principles.

**Methods:**

Data were collected by semistructured, in-depth qualitative interviews (N=9) of young people with type 1 diabetes aged 18-21. Interviews were transcribed and loaded onto NVivo for theme identification. Data analysis was undertaken during initial interviews (n=4) to locate potential ideas and enhancements for technical development. Latter interviews (n=5) assisted in the iterative sociotechnical design process of the development and provided additional developmental ideas.

**Results:**

Six themes were identified providing an understanding of how participants lived with and experienced their condition and how they used technology. Four technological suggestions for improvement were taken forward for prototyping. One prototype was developed as a clinically approved app. A number of ideas for new mobile apps and enhancements to currently existing apps that did not satisfactorily cater to this age group’s requirements for use in terms of design and functionality were suggested by interviewees but were not prototyped.

**Conclusions:**

This paper outlines the nonprototyped suggestions from interviewees and argues that young people with type 1 diabetes have a key role to play in the design and implementation of new technology to support them and improve HRQOL. It is vital to include and reflect on their suggestions as they have a radically different view of technology than either their parents or practitioners. We need to consider the relationship to technology that young people with type 1 diabetes have, and then reflect on how this might make a difference to them and when it might not be a suitable mechanism to use.

## Introduction

The World Health Organization has identified the treatment and care of diabetes as a major challenge for health care systems worldwide [1]. In the United Kingdom, the National Health Service (NHS) spends at least £3.9 billion a year on diabetes services, with around 80% spent on treating avoidable complications [2].

Type 1 diabetes occurs when the body produces no insulin because of autoimmune destruction of the pancreatic cells that normally produce it [3]. It can occur at any age but usually develops before the age of 40, often during teenage years. Patients with type 1 diabetes need to take insulin injections for life and, in order to reduce their risk of developing complications, must ensure their blood glucose levels are sufficient to balance their insulin doses, diet, and activity. They must also carry out regular blood testing. The primary diabetes outcome is glycemic control, as measured by a blood test (glycosylated hemoglobin or HbA1c) that indicates average plasma glucose for the previous 2-3 months [4]. Poor glycemic control has been related to short-term consequences such as hypoglycemia and diabetic ketoacidosis, as well as serious health consequences later in life [4]. Completion of recommended self-care tasks is considered critical to glycemic control, with the primary tasks that help maintain control—such as monitoring blood glucose levels, injecting insulin, and dosing insulin according to meter results or other factors—needing to be carried out several times per day, often around mealtimes in different contexts and locations [4]. Type 1 diabetes is the most common form of diabetes in most parts of the world, although wide variations exist between the incidence rates of different populations [5]. In the United Kingdom, it accounts for 10% of all people with diabetes and 90% of young people with diabetes [6].

In November 2012, a UK Public Accounts Committee (PAC) published its report on the management of adult diabetes services in the NHS, stating that the standard of care for diabetes in England was “depressingly poor”, causing unnecessary deaths and disabilities. This report followed critical studies on diabetes care from both the National Audit Office [7] and Diabetes UK [8]. The PAC Chair stated [2] that although the Department of Health had set out clear minimum standards for diabetes care—including nine basic checks for the early signs of avoidable complications—fewer than half of people with diabetes were receiving all nine tests. Variations in the level of progress across the NHS also meant that there was an unacceptable “postcode lottery” of care, whereby quality of care varied dramatically around the country. In November 2012, members of the House of Lords debated the management of diabetes services in the NHS following the PAC report. Lord Harrison, who had lived with type 1 diabetes for 43 years, stated his biggest concern was that the onus of care and of making important decisions was becoming the sole burden of the individual with the condition [9]*.*

Education about long-term complications via access to information could help patients with diabetes empower themselves to manage their condition more effectively, thereby reducing complications. It has been acknowledged [10] that better information on the ways in which social support operated was vital for enhancing diabetes patient self-care, insuring adherence to advice, encouraging lifestyle changes, helping to improve outcomes of care, and increasing personal freedom.

Lamb’s article on integrating technology into adolescent type 1 diabetes care highlighted how metabolic control varied with age [11]. Results from this study showed a progressive rise in HbA1c values throughout adolescence, peaking through the ages of 18 and 22, before falling again in early adulthood. Lamb considered a number of factors to be at work that could affect an inability to control HbA1c during late adolescence, and for these reasons, suggested that it was not surprising that metabolic control deteriorated while the incidence of acute complications such as diabetic ketoacidosis increased during adolescence [11]. Walker [12] defined health-related quality of life (HRQOL) as the level of well-being and satisfaction associated with an individual’s life and how this was affected by disease, accidents, and treatment.

To date, there exists little data analyzing how young people with type 1 diabetes make use of Web and mobile technology and its impact on their HRQOL. Franklin’s [13] study was the first randomized control trial (RCT) that explored the impact of SMS text messaging (short message service)-enabled behavioral support, with intensive therapy in a young age group. However, the study made no mention of engaging with the target audience to discuss what they would like to use, in order to influence the hypothesis of the study. Similarly, Pena’s [14] cross-sectional Web-based survey of parents with children who had diabetes focused on adults, again lacking a focus on the concerns of the young people, which were neither addressed nor included. Reporting on an Internet-based self-management intervention, the authors [15] stated that their research was the first trial of an Internet program to improve problem solving in adolescents with type 1 diabetes. However, the study again made no reference to having asked young people their opinions during the design of the intervention. In 2012, an article [4] highlighted how little was currently known about how young people used mobile phones for diabetes and as yet only a small proportion of apps available had been the subject of any research [16]. This view was most recently reaffirmed [17] in research that noted a lack of literature available on strategies to promote greater engagement of youth in behavioral interventions for type 1 diabetes, with even less information on the use of the Internet and mobile technologies for minority and low-income youth.

This poses the question as to why more health professionals, researchers, and technologists have not approached this age group for their opinions and suggestions. Why have these views seemingly been ignored? Is it a trust issue or perhaps professionals consider themselves more knowledgeable about the condition and its effects than the actual people with the long-term condition? There is a real need to explore how young people with type 1 diabetes relate to their condition; how they use and interact with technology and the Internet in health and non–health-related situations; and what they think would be useful in new health-based technological innovations. This could be achieved by talking to them and asking them for their opinions and suggestions. The research question was: *How do young people with type 1 diabetes interact with technology in their lives and in relation to their condition and how can their views and experiences inform the development of a patient-centric mobile health app?*

In recent years, qualitative research methodology has become more recognized and valued in diabetes behavioral research [18] because it helps answer questions that quantative research might not, by exploring patient motivations, perceptions, and expectations. Lifeworld studies concerning diabetes [19-21] have also started to appear more often in research literature. The study aimed to gain a deep understanding of the perspective of young people with type 1 diabetes and connect with their views by building a picture of their everyday experiences with the condition and how they used technology both socially and for health, as influenced by approaches from qualitative research in health care such as the lifeworld. Then, aiming to integrate this perspective within the creation of a mobile or Web tool influenced by these opinions, which would help to improve an aspect of HRQOL by using humanizing sociotechnical principles [22] during the design and build.

## Methods

A generic qualitative approach was adopted that would allow for the development of a breadth (allowing participants “maximum freedom in expressing the range, scope, and boundaries of the complex experience” [23]) and depth (further exploration of specific events and experiences in the participants’ lives [24]) of understanding regarding the nature of the studied experience. Recruitment was conducted at a district hospital located in south west England (SWDC) and a local university, with data collected by qualitative interviews with young people with type 1 diabetes aged 18-21. Although the clinic had children under 18 attending, the focus was on older members as this alleviated the need for parental consent. The upper limit was set at 21 years as this was the age participants no longer attended the Young Person’s Clinic on a regular basis. The sampling strategy utilized a nonrandom convenience sample, as selection was from participants who had type 1 diabetes within the population definition. The sampling strategy was purposive (nonrandomized). Participants were considered eligible if they had type 1 diabetes, were 6 months post diagnosis, were within age range at time of recruitment, and were fluent in English.

The design used in-depth, 1-hour, semistructured interviews. Semistructured interviews are used when the researcher knows what questions they want to ask but does not know what answers to expect [25]. Question stems are usually asked in the same order during each interview and responses to open-ended questions can then be probed, so that the interviewee has the freedom to respond as they wish [25]. In-depth interviews are typically used when seeking information on individual, personal experiences from people about a specific issue, to capture their own voices and stories [26]. It was decided to progress with individual interviews rather than use focus groups, as SWDC staff indicated that a young age group with a mix of male and females would not produce good quality results. It was also noted that this age group had a preference for talking individually and that some interviewees might find talking about particular aspects of their condition and lifestyle embarrassing or stressful within a group setting. Finally, as Morse [25] noted, focus group opinions are offered publically, so they might not always reflect the interviewee’s actual response.

A semistructured interview guide [27] was developed. Some broad questions and areas of interest were prepared beforehand, but it was recognized there was also a need for improvisation during each interview, based on anticipated and unanticipated responses. The general purpose of each interview was to discuss, in detail, specific topics related to the interviewee’s knowledge and its relevance to the research question and objectives [28], which were to explore young people with type 1 diabetes perspectives of their day-to-day lives and how they made use of Web and mobile technology and to identify from these views and experiences, how they used technology (if at all) in relation to their condition and treatment. The core focus of each interview moved from the interviewee’s first mobile phone and the historical timeline of different phones they had owned, to questions about the history of other mobile and computer technology they used. This usually led to a discussion about their usage of different Internet software and social media tools. This was then followed at an appropriate point by a question about their diagnosis date, which might then lead to talking about their diagnosis and how they had used technology (if at all) since then. This could then lead into a discussion of different aspects of their day-to-day life with diabetes; their experiences of the clinic, GP surgeries, and other health services they encountered; experiences socially, at school, at home, and at work; how they coped with and used technology related to diabetes; and, if they had used any health-related apps. When discussing any problems they had experienced, we would then explore what ideas or enhancements they might have for something that could improve that aspect of their lifestyle or others, and whether a technical solution might be of any help.

In total, (N=9) interviews were conducted (m=2 and *f*=7), transcribed, and loaded onto the qualitative data analysis tool, NVivo. The interviews were then analyzed to gain a deeper understanding of the perspective of the young person with type 1 diabetes and to construct a picture of their everyday experiences. Credibility refers to the accuracy of information obtained during a study and is maintained via triangulation of data sources, methods, and investigators. One way that credibility was achieved was by prolonged exposure to the subject being investigated. By spending a substantial period of time in the clinic and observing the day-to-day activities and routines of its staff, it was possible to become immersed in the world of the clinic. During numerous visits there, the researcher was able to build relationships with practitioners, dietitians, and receptionists. By spending longer periods of time with interviewees, they were able to build their trust over the course of interviews. A further method of establishing credibility came from regular supervisory contact, with a requirement to satisfy the supervisory team that research procedures and ethical standards were being followed at all times and also to defend ideas, methods, and analysis during extended questioning on all aspects of this study.

## Results

Six main experiential themes were identified providing an understanding of how participants lived with and experienced their condition and how they used technology: (1) living with diabetes, (2) diabetes technology, (3) in the clinic, (4) obtaining information and support, (5) mobile technology, and (6) mobile apps and mobile health apps.

**Figure 1:**
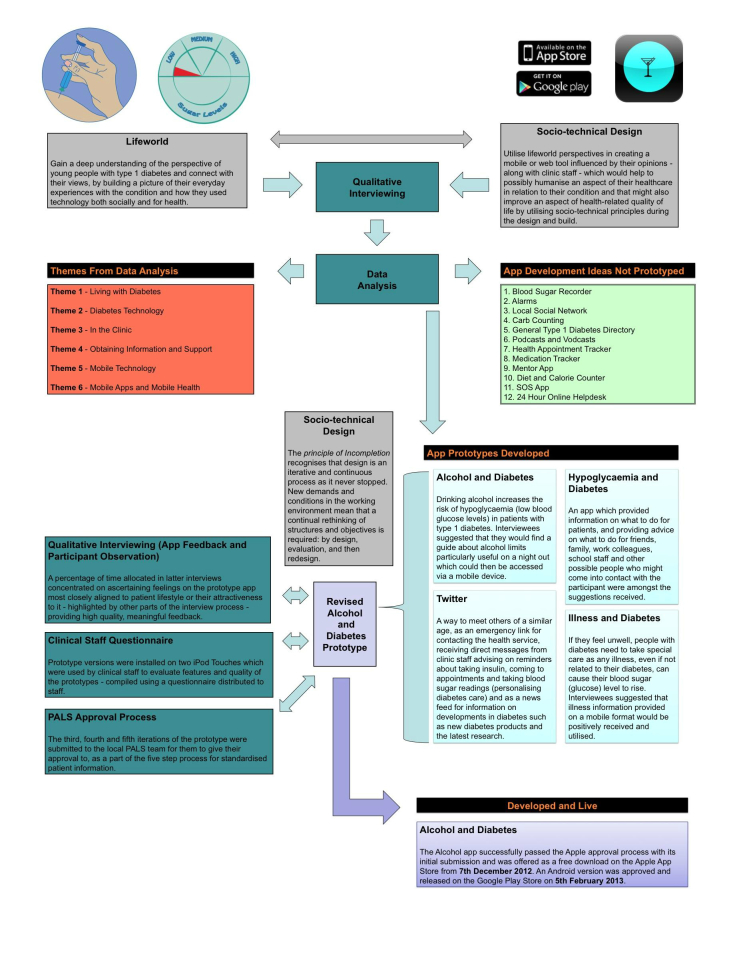
Methodological structure.

Besides providing an understanding of their day-to-day experiences and how type 1 diabetes affected their HRQOL, interviewing enabled the identification of possible ideas for the development of prototype mobile apps. The suggestions needed to meet SWDC goals, reflect interviewee requirements and comments, and follow local trust guidelines (eg, patient data were not allowed to be recorded). By asking for their suggestions, in collaboration with the clinical team, we were able to focus on four ideas for prototype development [29]. Three of these were created in prototype, with one subsequently chosen by later interviewees (n=5) to be taken to final development [30]. The prototype development used sociotechnical design principles [31]. This approach has recently started to re-emerge in health literature, with examples within diabetes research using it as a means of collecting data for systems designed for both staff [32] and patients [33]. A key characteristic of sociotechnical thinking lies in highlighting the importance of developing new ways of working that significantly meet the needs of clients (patients) and users (service providers) [34]. This developmental work and the final app produced could help contribute to new knowledge and understanding of young people’s requirements and concerns, which could then improve their HRQOL. Additionally, a number of other innovative ideas and suggestions for enhancements were made for improving their lifestyle and making a difference in other areas of their lives affected by their condition—these ideas were suggested during the interview process but not taken forward for prototype development (see Figure 1).

### Blood Sugar Recorder

Capillary Blood Glucose Monitors (CBGM) are capable of holding a large number of historical readings and can sometimes be linked to a computer to transfer data. However, this cannot currently be performed wirelessly. This compares to cloud systems like Apple’s iCloud, where data is transferred seamlessly between devices. The constant proximity of the mobile phone to the user makes it an attractive option for use as a recorder. If it could be linked to the data cloud, information might then be accessed and displayed across multiple devices.

Cause again that’s something then you’re looking at that’s more visual, that you can see oh ok, maybe it’s not as good as I thought, whereas just doing it, doing your sugars once every day and just seeing the numbers, just seeing it on a chart, seeing how it is over a period of months, that could be quite…

T1-QOL-05

So maybe an App that could give more advice,’cause coming to the hospital’s really good, like they do set you targets, like I came before Christmas and they set me the target of just doing er, my sugars once a day which I’ve stuck to.

T1-QOL-05

And then, the personal notifications, I wondered if you could put other things up like erm, like your insulin, or what you’ve eaten. Sometimes they weigh you, measure your height and things. Maybe you could put that on there.

T1-QOL-09

This could then be taken to a clinic to show to staff or also be used for creating historical graphs to pinpoint times of good and bad control, making it easier to see where somebody was going wrong by highlighting higher than normal readings. Historical graphing could also provide a useful incentive for improving control, as it would allow the ability to immediately call up and view the data at any time. This may also be more accessible to a user with pleasing figures and colors. Historical readings are available on newer CBGMs, but they are not as accessible or as attractive to view when compared to a digital display. Personalized goals and targets could also be set, which could then be flagged if they were met or missed. A more interactive diabetes assistant could also be provided to tell users when they were doing well or not. This may be more effective because interviewees view their phone as a friendly device, not automatically associated with their condition [11], as opposed to their CBGM, which might be viewed as more specifically linked to their condition—similar to how interviewees differentiated Facebook usage for health and personal use.

Functionality might also be provided to remind the user to take a reading by using the push function of a mobile device, which could then display alarms and reminders as pop-up SMS text messages. This could also be tailored to include personalized information about insulin, weight, food intake, height, and other useful data. However, some users might not wish to enter the data twice, as it is already on their CBGM, or they might find the task of inputting figures from a blood sugar reading onto a mobile device just too bothersome—even if the task was simple and easy to perform. Some of these suggested features appear on the iBGStar [35], which is the first CBGM that can be used on its own or connected directly to an Apple iPhone or iPod Touch to display, manage, and communicate diabetes information, and which also works in conjunction with a specifically written self-management app. However, no research is available on how this CBGM and app have been utilized to date. It is only available to buy in the United Kingdom (rather than offered for free through clinics), so alternative methods of support could be offered via a free app that worked across a range of different CBGM rather than locking the individual and the clinic into one particular device with its associated costs.

### Alarms

Some interviewees had already used the alarm clock function of their mobile phone to remind them to take insulin or to obtain a blood sugar reading. Expanding on this idea and once again applying the principle of utilizing push technology on a mobile device, it would be relatively straightforward to produce a simple-to-use alarm app (see Textbox 1).

This could allow users to set up multiple reminders on insulin, blood sugar level checking, and eating, thus helping individuals to be persuaded or even nagged into doing something specific. However, some users might simply choose to ignore the alarms, which could be addressed by changing the tone of the information contained within the message, received over a longer time period. While acknowledging that some of this functionality already exists within apps like OnTimeRX Pro [36], interviewees had been unable to locate anything that satisfactorily catered to their requirements or their preferences for design and functionality.

**Comments on alarms ( I: = interviewer speaking / P: = participant speaking / [T1-QOL-XX] = participant interview number ).**

I: *Right and does that mean you’re occasionally forgetting to look or you might be missing meals or…*

P: *Missing injections and or given too much insulin*

[T1-QOL-01]

I: *Yeah. Ok, so do you think having a reminder or a something on there that sort of nagged you…?*

P: *Yeah, definitely. I definitely need a push with it…*

[T1-QOL-05]

I: *A little box to say “Have you taken it yet?”, “Why haven’t you taken it yet?”*

P: *Yeah (laughs).*

I: *You need to take it now!*

P: *Yeah (laughs).*

[T1-QOL-09]

### Local Social Network

The belief that users of this age group were happy to share every aspect of their personal life on Facebook (including health) was contradicted. Some interviewees wanted to use Facebook only for talking to friends about their life outside of type 1 diabetes, rather than using it for diabetes-related searches and communal group discussions. It is also important to consider how quickly popular opinion in this age range changes concerning different brands and products. Indeed, it seemed as if the amount of discussion in the media about certain social media tools like Twitter was in some cases harming its ability to attract this age group toward using it. Some mentioned the problem of having too much information being constantly tweeted at them by too many people—similar to the Facebook phenomenon of too many people being available to connect with, causing them to exert tight controls on what they looked at and where they visited online.

Consequently, the idea of a small personalized social network was discussed by interviewees (see Textbox 2) in the style of a local, private Facebook or Twitter community, which might encourage communication between clinic attendees and help them to make new friends with the same condition—quite difficult for this age group—in order to address the isolation they sometimes experienced at that age. This could then link into wider regional, national, and international networks. The forum could be split into private and professional areas, with the private area being used for making friends and venting personal opinions in a secure environment with, notably, people of the same age. Establishing this private forum that clinic staff could not access, but which had their patronage, rather than setting up a group administered within Facebook, might even encourage wider participation (interviewees suggested this point). The other part of the forum might be managed by clinic staff, although cost could be an issue, and be accessible to patients for their queries and feedback. Private queries—which users might not want clinic staff to see for whatever reason—could be made within the secure private space. Again, easy access via a mobile phone might encourage some to more actively participate in this type of discussion. Previous research by Pew has shown that wireless connections are associated with deeper engagement in health-related social media, with mobile Internet users more likely than those with tethered access to post comments and reviews online about health and health care [37]. TuDiabetes [38] was praised by one interviewee as being a particularly friendly communal environment that raised general awareness and provided other information related to their condition, as well as for ease of posting personal queries in the discussion forum. However, only 1 out of the 9 interviewees knew about this community, which suggests that awareness of this forum is not currently high with this age group in the United Kingdom.

**Comments on local social networks.**

I: *if we wanted to keep it to [clinic location] would you be happy if it went further say if it was used nationally or would you really want it sort of kept specific to [clinic location]*

P: *Yeah, it could be used nationally, but then if you same, in sense under it have like separate departments where you could go to*

I: *Almost like, little mini networks...*

[T1-QOL-01]

P: *But I would definitely use, um, a smartphone to do more for my diabetes with it, even if there was just some specific forum where people could talk…*

[T1-QOL-03]

P: *maybe like erm, a forum, erm, which had erm, both people with diabetes and medical professionals who were part of the forum so if you posted something like if you had a query, you could get feedback from people who possibly have gone through it and from like a medical point of view as well*

[T1-QOL-04]

### Carb Counting

While positively viewing Carbs & Cals [39] as a good quality mobile app for diabetes information, interviewees suggested that it was not a perfect product in every aspect. They responded positively to the ability to access more localized information on a mobile platform (see Textbox 3) for their age range and younger, remembering back to just after diagnosis. They valued an app covering items that they had not eaten before, highlighting the importance of a continually updated food database; an app that also provided a better UK perspective on foods, including more detailed information on certain UK restaurants, takeaways, and fast food establishments, more akin to what this age range were actually eating; and one that provided a more practical guide to UK-specific snacks, crisps, biscuits, and chocolate bars. This information could then be presented in a searchable, indexed format within the app, providing a more portable way of carrying around large amounts of information. Or, for simplicity, this information could be made available as a specially tailored eBook or PDF, instead of an app, so that it could be accessed without the need for a constant Internet connection.

**Comments on carb counting.**

I: *… say you put that paper booklet of carb values on there er, would you use that? Sort of call that up?*

P: *Yeah, probably. Because every now and then you come across something that you haven’t really eaten before so it’s quite good to be able to reference somewhere, what, how many carbohydrates are in them…*

[T1-QOL-09]

P: *…I quite liked looking at all like the [fast food chain] and restaurant ones, I think they’re the most difficult, because you don’t see the ingredients…*

I: *Hmm.*

P: *…so I think an App like that would be useful…*

[T1-QOL-06]

P: *But rather than carrying around a book, if you’ve got your phone with you, and you’ve got the app that’s the carb counter, the only thing is, most apps require Internet or like a decent signal to get it. It would be better if you got one that you downloaded as maybe a PDF file or something that was constantly on your phone as an e-book or something rather than an app...*

[T1-QOL-08]

### General Type 1 Diabetes Directory

When a young person is diagnosed with type 1 diabetes, they need instant access to a vast array of information concerning all aspects of their lifestyle. This is also applicable initially to parent caregivers. Interviewees suggested a general type 1 diabetes app directory to hold information on a number of topics (see Textbox 4). This might include a welcome message to be read in their own time, once they had been initially diagnosed, responding to some of their main fears and concerns in a sensitive, personalized style, and also featuring ways they could deal with some of the most challenging aspects of the condition. In addition to containing the contents of the generally informative patient education pamphlets offered by clinics, which this age range seemed to either throw away or lose and then only wanted to access again when they had a specific query, this directory could also include the specifics of how type 1 diabetes occurred rather than on the mechanics of insulin. It might also provide a tip sheet written for other parents and friends, which could be emailed to them as a PDF prior to a sleepover (like the parental handout that one interviewee’s mother had created for them) and could offer more lifestyle-focused information such as exercise, nights out, and alcohol. It would need to be updated regularly to provide the latest information, such as when new policy was formulated on areas like diabetes and driving laws. This would have the advantage of providing up-to-date clinically validated information offered for quick and easy access to users at a particular moment, rather than their having to trawl through different websites.

**Comments on general type 1 diabetes directory.**

P: *…if it was kind of like erm, an App which had like, like a contents like, Diabetes, erm, Alcohol, sorry, illnesses, things like that and you could quickly go and it had like Q&A’s that would be really handy…*

[T1-QOL-04]

P: *If it was a straightforward, easy to use, I’ve just done exercise and my bloods have gone high. It would take you to the sports page and give you an explanation why. That would be great…*

I: *Yeah*

P: *You know, if it was, if it was well-organized and um, sort of sub-sectioned so that you could find your problem within the space of maybe 5 minutes, rather than an hour...*

[T1-QOL-08]

### Podcasts and Vodcasts

Podcasts were positively received as a potential source for distributing and receiving engaging, good quality, useful information (see Textbox 5). Sometimes this was mentioned as being preferable to reading the information. Although podcasts can be made available for download directly through Apple’s App Store or via a website, interviewees preferred they be made available directly through an app to save time when accessing them.

Participants were mostly happy to view the information, such as via a vodcast, rather than listen to it. They preferred a less formal style than traditional informational messages from health services, either encouraging users on eating healthily for example or by offering a more serious tone if required. A final important note to consider was the type of presenter; a more professional one might garner the best results and engage the most users in the message being given to them.

**Comments on podcasts and vodcasts.**

I: *…thinking back to since eleven, if there was podcast information on particular subjects. Is that something you might listen to?*

P: *Yeah I probably would have listened to it, because I don’t really like reading and things so, listening to it would have been much better…*

[T1-QOL-09]

P: *So, I think I would be interested in watching YouTube videos if it was sort of educational. But I wouldn’t want to watch them if it was just somebody talking about…*

I: *Ok.*

P: *…you know like a vid- blog or something, er, not a blog a, where they record themselves and talk about their own thing. I don’t think I’d watch something like that...*

[T1-QOL-06]

### Health Appointment Tracker

The ability to monitor and book appointments over a mobile device via an app was also suggested (see Textbox 6). This app could also feature the ability to immediately record information obtained on the day of the appointment, such as the eyes and feet, which could then be accessed historically by the user.

The app could feature more generic recorded data functions than the idea suggested for recording blood sugar levels but might also have options for ketones and other general information. It would be possible to program this app so that the information entered could be customized and personalized for each user, enabling them to configure it to meet their needs more effectively. However, it should be noted that even within this age group, there were still some young people who preferred using paper rather than digital means for recording information and that some of this functionality already existed on the Diabetes Tracker app [40], although it did not appear to have been well used by those interviewees who had downloaded it.

**Comments on health appointment trackers.**

P: *…and then you have your eyes, and then you have your feet, and all things like that, so maybe, erm, something where you could put in when your last one was, what the results were, when your next one was…*

I: *Ok*

P: *…and when you’re next due one…*

[T1-QOL-04]

P: *Yeah, just write the appointments, I mean I had a letter with um, recently and I’ve booked it and I have put it on here as well (on phone) but I, I just find it easier to have on paper in front of me…*

[T1-QOL-06]

### Medication Tracker

Although not required by all interviewees, the use of a medication tracker was also suggested as a method of improving daily HRQOL (see Textbox 7). This app could allow the user to simply tick a box once a medication had been taken, perhaps with a date and time stamp attached, and could be customizable to include a number of different medications. Using mobile phone push technology, this could also include pop-up reminders to be configured based on the different medications and the times they were due to be taken.

These warnings could increase in severity if not acted upon, similar to the alarm app idea. Again, this functionality does already exist on other apps [36], but interviewees had not located something usable or found existing products to be designed with them or their condition in mind.

**Comments on medication trackers.**

P: *…I’ve got levemir, I’ve got a couple of other daily medications as well, just be able to sign and say push, yes I’ve done my levemir today and if I, um, ’cause sometimes I do my levemir and I’m like, “Have I done my levemir today?” and have this horrible panicky feeling that I might not have done my levemir but I don’t want to do more, do it again if I’ve done it already…*

[T1-QOL-02]

I: *Yeah, so just a little tick thing then, that would sort of say like, right, I’ve had this and…*

P: *Yes, possibly, probably one that could be customized to a number of medications as well…*

[T1-QOL-02]

### Mentor App

Some participants expressed a desire to be able to talk to people younger than themselves who had just been diagnosed, to act as their mentors and help them work through the various aspects of being diagnosed with type 1 diabetes, based on their own experiences post diagnosis. This could include the use of podcasts or vodcasts, where they discussed particular situations, including times when they had been feeling particularly low and how they had gone about counteracting those feelings. This would not aim to preach, but rather offer supportive understanding from a different perspective, away from their usual group of family and friends. Alternatively, they could offer advice from within a forum on particular questions that other young people just diagnosed might have.

I’d like to be like, be able to talk not to, yeah like younger people who are like fourteen, fifteen, sort of, who don’t really know where they’re going and they can talk to someone who’s actually been the worst diabetic, and who probably is the worst diabetic, you know, but who can see the other side of it as well like I’ve, I’d like to be able to talk to all different people about that I suppose…

T1-QOL-03

Because I like to be able to help the people that don’t have such good control

T1-QOL-06

The most positive aspect of this suggestion is that it shows how young people with type 1 diabetes are keen on sharing their knowledge, providing information and support, and helping out others in a similar position to themselves or who were just beginning their post-diagnosis journey. There does not appear to be the right set of circumstances or opportunities for this to currently occur according to interviewees.

### Diet and Calorie Counter App

While acknowledging that there were a number of existing dietary and calorie counting apps already available, such as My Diet Diary [41], the limitations mentioned with them made the design of a newer, more effective app attractive to interviewees (see Textbox 8).

The new app could help take some of the guesswork out of calculating calorific and carbohydrate values and allow users access at convenient times and in different locations. The major factor for something like this was to be able to replace the vast amounts of literature that would need to be carried around on visits to an external location or restaurant. Enhanced functionality might also include offering advice on healthy eating, weight recording, historical tracking, and offering encouraging messages via SMS text alerts, podcasts, or vodcasts to make the process of controlling weight and eating healthily more attractive and supportive.

**Comments on diet and calorie counter apps.**

P: *I have the books at home or in my flat, but I wouldn’t carry it around in a bag like this or something to go out for a meal or I’d just try and guess work how much carbs I was eating but whereas if you can just flick it onto, on an app it would be much easier wouldn’t it? I think it would be good for accessing it really...*

[T1-QOL-03]

I: *…maybe a little sort of weight related in terms of um, encouraging you to um, sort of put your readings in to keep…*

P: *Yeah*

I: *…to keep going with that, when you’ve started…*

P: *To stick to healthier eating, yeah.*

[T1-QOL-05]

### SOS App

A free BlackBerry app, the Personal Guardian [42], allows a user to summon help with the press of a button in an emergency situation. Users send an SOS by triggering a silent alarm to call 911 in the United States (or any other number entered), in addition to sending an email, text message, or Twitter post with the user’s current location, using GPS technology within the device. Users do not have to unlock their phone or wait for the app to open; they simply hold down the convenience button on the side of their BlackBerry. Being able to provide a similar function to this app within a diabetes environment would offer several similar benefits (eg, in the event of a hypoglycemic episode) and might also offer improved HRQOL solutions for other conditions like epilepsy. This was viewed as being a very useful solution because young people with type 1 diabetes were reluctant or often forgot to wear medical alert bracelets as they got older: “…because if you did collapse, you know, they’re going to look for your home number or someone to contact it might be really useful to say [laughs nervously] in the event of an emergency you know, just click on that thing on the iPhone or a BlackBerry...” [T1-QOL-03]

### 24-Hour Online Help Desk

Another beneficial suggestion, though appreciably more difficult to maintain due to the high cost implications, was the production of a 24-hour online help desk that could be accessed via an app, SMS text message, email, Twitter, or real-time chat facility. The challenges may include funding this idea, along with the complexities of the design and programming, rather than the use of specific communication media; any of the above suggestions would provide an improved service to help improve HRQOL. One interviewee noted: “In the event of an emergency, I mean if someone could just tweet on there ‘Help what do I do? So and so’s got a nose bleed and she’s diabetic’ or something like that you know”. [T1-QOL-03]

It is more likely that this age group would want to engage with these sorts of media rather than older users based on current literature [43,44]. But, by providing this service at a younger age, it could be argued that over time health services would actually benefit, saving money by decreasing the number of complications experienced by this age group, which in some cases led to their hospitalization [2].

## Discussion

This research highlights that there are many ways in which the HRQOL of young people with type 1 diabetes could be improved, through the design and implementation of new technological innovations and enhancements that use Web and mobile technology. This research tells us that there is a need to consider three factors before anything is actually developed: (1) considering young people’s relationship to technology, (2) reflecting on how this might be able to effectively make a difference to them, and (3) considering when it might not be a suitable mechanism to use.

### Young People’s Relationship With Technology

The age when most interviewees obtained their first mobile phone was quite young in comparison to previous generations—on average, at the start of teenage years, although some interviewees had obtained them even earlier, in some cases, from the age of 9 or 10. Therefore, from a relatively young age this generation has viewed their phone as a constant companion, accompanying them at play, through school, university, and then work use: “because I wasn’t carrying the book around with me, whereas with my phone…it’s always with me wherever I go”. [T1-QOL-05]

It appears that newer smartphone devices have gradually started to replace other electronic media that interviewees owned like MP3 players, laptops, cameras, and paper-based systems like diaries. As the capabilities of smartphones have increased, they can be viewed as ever more attractive options for use in relation to type 1 diabetes. Indeed, 60% of 16 to 24-year-olds in the United Kingdom now use a mobile phone to access the Internet every day [43], while in the United States, 42% of mobile owners aged 18-29 have looked for health and medical information on their mobile devices [44]. Because this generation of users has become more attached to their mobile device, there are important implications on how future education, awareness, and management of type 1 diabetes could be changed or integrated with technology. For example, it has been noted [45] that the majority of adolescents wished to communicate only by SMS text message for follow-up post education, highlighting the need for health professionals to adapt to the lifestyle and mechanisms of communication adopted by today’s adolescents. Literature suggests that there is a thirst for new technology to be applied in the care of type 1 diabetes and on the rare occasions when young patients have been asked [46], they were keen on trying new solutions, although there remain doubts as to whether technology can effectively help them in all aspects of education and self-management [11].

### How Technology Can Make a Difference

In some cases, the closeness of technology to the young person had already led to it being used innovatively in relation to their condition, such as using alarm functionality on a mobile phone. It is important to note that some users would be more likely to engage with diabetes-specific, app-related technology if cost were not an issue. The main benefits mentioned by interviewees of using diabetes apps tended to focus on their ability to replace paper-based information, like logbooks and books, which they previously had to carry around with them, in addition to their other diabetes equipment. However, there was a feeling that current apps, although helpful, were not actually worth using. This could be due to the cost, flexibility, design, and usefulness of the apps themselves, or the lack of a cohesive guiding framework for using them, which could involve the clinic in some way. For example, if a CBGM automatically logged blood glucose, what was gained by interviewees having to additionally enter it themselves on their phone? The lack of a decent quality app for blood glucose monitoring was mentioned by one participant directly while others lamented the lack of UK-centric apps; the dietary values on those available were slightly different, making them difficult to use effectively. This was a comment also directed at some of the data held on the Carbs & Cals app [39], even though the app itself was well regarded. Participants could see the value of accessing up-to-date information via some form of technology like Twitter [29] as a means of providing them with support and information, such as increasing awareness of information on new CBGM about to be released and other technological innovations, highlighting news on scientific advances, and helping with support immediately after diagnosis.

Technology could also be used to ease the transition between different clinics and prevent issues such as the lack of notification of changes in guidance [29]. This could help minimize the problems of receiving differing, seemingly contradictory, advice from different parts of the health service. Technological enhancements were suggested for the development of health apps that might be useful to them in the areas of Twitter [29], illness and diabetes [29], hypoglycemia and diabetes [29], and alcohol and diabetes [30]. These were in addition to the many other ideas and suggestions they had for improving their lifestyle and making a difference in other areas of their lives affected by their condition, which were not taken forward to development (see Results).

As can be seen from these suggestions, there are a number of areas where new technological solutions might help to bridge a gap and offer new opportunities to improve HRQOL for this age group and condition. It is a surprising aspect of this research that there were so many areas mentioned that have yet to be adequately addressed, either not existing at all or existing in an inadequate or unsatisfactory product for this age group. It is more surprising in light of the current poor performance of UK diabetes care and the length of time these technologies have now been around for: “There is apps on here but I’ve never used them because they don’t seem that good. Just seems like someone in the back shed’s made them”. [T1-QOL-01]

### Considering When Not to Use Technology

There was a strong feeling expressed by interviewees that although the condition, type 1 diabetes, might be the same, the experience of living with it was completely different for each individual. Just as each person had their own unique perspective and a completely different personal experience of living with type 1 diabetes, so they also required different approaches and suggestions based on their own personal preferences toward technology: “no one, no two people are the same with diabetes, everybody is different”. [T1-QOL-08]

This means not assuming that everybody in a particular age group will automatically adopt any new form of social media or technology. It is notable that not all interviewees were drawn to engage with new products if they did not directly appeal to them in some way. For the majority of users, from a mobile perspective, apps were quite a new development even though launched some time ago, and some interviewees had not made much use of them. One of the reasons for this was financial; users were reluctant to try them out because they were frightened about the implications of creating an account or they were reluctant to pay for an Internet connection as a part of their mobile contract. The general theory concerning Web 2.0 and social media suggests that it facilitates and encourages use and collaboration. So, it is in stark contrast that this particular age group does not always feel inclined to use it as a mechanism for obtaining information and support for their condition. It might be that aspects of their character or personal feelings limit them from interaction in online environments with people they do not know. Or they might have conditions precluding certain areas or functions of online activity, like Asperger’s syndrome. They may not wish to share information about their condition online—the technological equivalent of Williams’ [47] findings on how diabetes is sometimes kept separate from social identity. Aspects of technology use that might be diversive; use the wrong medium to spread a health message; try to manipulate something entertaining into something educational without clear education, explanation or support; or exclude through cost or software system could negatively impact on any technological enhancement implemented. They need to be given clear and careful consideration before any solution is developed.

### Limitations

The main limitations of this study are that it was not able to test the impact of the innovative tool that was created [29-30] and that more of the other suggested enhancements were not able to be taken forward for development (see Results). However, future research projects can help to address this, by measuring the impact of the created app locally, nationally and overseas using a validated HRQOL measure. Additional health apps based on this research approach can also be designed, tested, built and implemented using the same approach and then be subsequently measured for any positive or negative impact on HRQOL.

### Conclusions

We need to consider in depth the relationship to technology that young people with type 1 diabetes now have and then consider how this might make a difference to them. But we also need to decide when it might not be a suitable mechanism to use. By reflecting on these areas, any technology to be created will be much more usable and suitable for the target users. Reflecting on the tenets of good sociotechnical design related to the creation of new or enhancements to existing health apps, democratic and participative communication and decision making must always be available to give users a voice [22]. Future developments concerning the use of mobile phones and health apps should reflect and focus on how this generation has become accustomed to using them and where they might best fit best in a health context - acknowledging that this will not naturally be successful in every circumstance. This will be a key way to address a broad range of enhancements to improve HRQOL that will be used regularly and make a real difference. For example, a new CBGM app could be tailored to include personalized information about insulin, weight, food intake, height and other useful data, without replicating the functionality of existing CBGM that already have the ability to record these data. Stepping back and taking a more practical, logistical approach to some of the problems users experienced, a tip sheet written for other parents and friends could be emailed to them as a PDF prior to a sleepover. Calorie and carbohydrate counting material could be provided as a specially tailored e-Book or PDF instead of as an app, so that it could be accessed without the need for an Internet connection via a simple and quick indexing system. Another options would be enhancing (based on user requirements) or using existing mobile phone technology in new ways to harness improvements to HRQOL, such as using push technology for pop-up reminders about insulin injecting, blood sugar level checking and having something to eat (but in a more attractive and interactive way for younger users).

The need is there and has been highlighted—we just need to create the proper technological solutions that this user base is asking for. The World Health Organization estimates that more than 80% of diabetes deaths occur in low- and middle-income countries and projects that diabetes deaths will increase by two thirds between 2008 and 2030 [48]. We hope that the dissemination of these innovative ideas and enhancements for possible mobile interventions, education, and support will offer ways to help reduce these figures. It is vital for policy makers, health practitioners, and technicians to take note of and reflect on these ideas and the issues that they raise. Helping to build an enhanced understanding of young people with type 1 diabetes and what they might use, what is not being provided to them in the format and design they require, and what they would like to see in future type 1 diabetes education and support could improve HRQOL and reduce the health care burden.

## Abbreviations

CBGM: capillary blood glucose monitor
HRQOL: health-related quality of life
NHS: National Health Service
PAC: Public Accounts Committee
RCT: randomized control trial
SMS: short message service
SWDC: district hospital in South West England
